# Phenotypical Diversification of Early IFNα-Producing Human Plasmacytoid Dendritic Cells Using Droplet-Based Microfluidics

**DOI:** 10.3389/fimmu.2021.672729

**Published:** 2021-04-29

**Authors:** Laura C. Van Eyndhoven, Eleni Chouri, Nikita Subedi, Jurjen Tel

**Affiliations:** ^1^ Laboratory of Immunoengineering, Department of Biomedical Engineering, Eindhoven University of Technology, Eindhoven, Netherlands; ^2^ Institute for Complex Molecular Systems (ICMS), Eindhoven University of Technology, Eindhoven, Netherlands

**Keywords:** plasmacytoid dendritic cells, droplet-based microfluidics, diversification, heterogeneity, interferons, cytotoxicity, stochasticity

## Abstract

Plasmacytoid dendritic cells (pDCs) are a rare type of highly versatile immune cells that besides their specialized function of massive type I interferon (IFN-I) production are able to exert cytotoxic effector functions. However, diversification upon toll like receptor (TLR)-induced activation leads to highly heterogeneous responses that have not been fully characterized yet. Using droplet-based microfluidics, we showed that upon TLR7/8 and TLR9-induced single-cell activation only 1-3% secretes IFNα, and only small fractions upregulate cytotoxicity markers. Interestingly, this 1-3% of early IFN-producing pDCs, also known as first responders, express high levels of programmed death-ligand 1 (PD-L1) and TNF-related apoptosis-inducing ligand (TRAIL), which makes these hybrid cells similar to earlier described IFN-I producing killer pDCs (IKpDCs). IFN-I priming increases the numbers of IFNα producing cells up to 40%, but does not significantly upregulate the cytotoxicity markers. Besides, these so-called second responders do not show a cytotoxic phenotype as potent as observed for the first responders. Overall, our results indicate that the first responders are the key drivers orchestrating population wide IFN-I responses and possess high cytotoxic potential.

## Introduction

Plasmacytoid dendritic cells (pDCs) are a rare type of highly versatile immune cells constituting an extremely promising therapeutic target for infectious diseases, autoimmune diseases, and cancer. Besides their highly specialized function to produce massive amounts of type I interferons (IFN-I), previous studies highlighted their multifaceted biology that goes beyond the scopes of viral immunity (1, 2). In fact, it is widely appreciated that pDCs can also exert cytotoxic effector functions (3). However, in pathological conditions, pDCs are often in a hypofunctional or aberrant state, with tumor-infiltrating pDCs correlating with bad prognoses (4–6). Therefore, it is crucial to understand how pDCs can be properly activated, thereby enhancing their antitumoral potential and enabling them to restore local immune responses (7–9).

Over the past years, studies have revealed that pDCs are heterogeneous, showing a wide range of diversification upon stimulation. Subsequently, multiple different pDC subsets, specialized in multiple different functions, have been described (10–13). One of the most recent examples is the diversification observed in bulk-activated pDCs for the markers CD80 and Programmed death-ligand 1 (PD-L1), giving rise to three stable subpopulations of which one is specialized in IFN-I production (14). Although these studies have been very informative, an improved fundamental understanding of pDC diversification is needed to reveal their true intrinsic behaviors and capabilities. Therefore, experimental approaches require not only single-cell resolution, but also single-cell activation in which all types of cellular interactions that average out individual cellular responses are excluded. Droplet-based microfluidics is a high-throughput technique that allows highly controllable single-cell activation by encapsulating individual cells in picolitre-sized droplets that function as tiny bioreactors (15, 16). Inside these droplets, cells receive their input while being excluded from any kind of interaction with other cells, thereby revealing their intrinsic behaviors upon single-cell activation. This change in approach revealed that upon single-cell activation only fractions of 1-3% produce IFN-Is, which is a phenomenon that has been observed in multiple different settings, for different types of immune cells, both *in vitro* and *in vivo* (17–21). Additionally, IFN-I priming revealed the crucial role of paracrine signaling in amplifying the TLR-induced IFN-I response by increasing the fraction of IFN-producing pDCs up to 40%, which comes to a halt upon the administration of neutralizing antibodies (16).

In this study, we set out to investigate the cytotoxic diversification of pDCs to a similar extend as observed for IFN-I production. More specifically, we studied whether the small fractions of early IFN-I producers express cytotoxic markers, since so-called IFN-producing killer (p)DCs (IKDC/IKpDCs) have been described in the past (22–27). PD-L1 and TNF-related apoptosis-inducing ligand (TRAIL) have been known for their role in pDC cytotoxicity [reviewed in (3, 7)], and were therefore, together with a functional IFN-I readout, the main focus of this study. Additionally, intracellular granzyme B was assessed, since this cytolytic molecule has proven its anti-tumor effects in a variety of pDC and NK-cell dependent killing (3, 7, 28). Up to now, all that is known about pDC cytotoxicity and IKpDCs has been tested only in bulk-activated pDCs. Therefore, to the best of our knowledge, we are the first to study the diversification of IFN-I production combined with the expression of cytotoxic markers in single TLR-activated pDCs.

## Materials and Methods

### Cell Isolation and Culture

Human primary pDCs were isolated from buffy coats of healthy donors (Sanquin), according to institutional guidelines and after informed consent per the Declaration of Helsinki. Peripheral blood mononuclear cells (PBMCs) were isolated *via* Lymphoprep density gradient centrifugation (Stemcell Technologies, 07861). The isolated PBMCs were washed thrice with phosphate-buffered saline (PBS, Thermo Fisher Scientific, 20012027) supplemented with 0.6 w/v% sodium citrate dehydrate tri-basic and 0.01 w/v% bovine serum albumin (Sigma Aldrich, C8532; A9418). To deplete monocytes, PBMCs were resuspended in RPMI cell culture medium (Thermo Fischer Scientific, 11875093) supplemented with 2% human serum (pooled; Sanquin), 1% antibiotics (penicillin-streptomycin, Thermo Fisher Scientific, 11548876), and incubated for 1 hour at 37 degrees Celsius in T150 cell culture flasks. Afterwards, non-adherent cells were collected while washing the cells thrice with PBS. Next, pDCs were isolated using magnet-activated cell sorting (MACS) by positive selection using the CD304 Microbeat Kit (Miltenyi Biotec, 130-090-532), according to manufacturer’s instructions. For purity assessment, a small sample was washed with PBS supplemented with 0.5% bovine serum albumin (later referred to as PBA) and stained for 20 minutes at 4 degrees Celsius using FITC-labeled anti-CD123 and APC-labeled anti-CD303. The pDCs were identified as CD123+CD303+ (average 92%, SD 4.4%, *n* = 12).

### Soft Lithography and Microfluidic Setup

Microfluidic devices were fabricated with polydimethylsiloxane (PDMS) base and curing agent at a ratio of 10:1 (Sylgard 184; Sigma-Aldrich, 101697). After proper mixing, the PDMS mix was poured onto a master silicon wafer and cured at 65°C for 3 hours. The surface of the devices was OH-terminated by exposure to plasma (Emitech K1050X), and were sealed with plasma-treated glass slides to yield closed microchannels. Channels were treated with 2% silane in fluorinated HFE-7500 3M Novec (Fluorochem, 051243). Droplets were produced with a three-inlet microfluidic device. Liquids were dispensed from syringes driven by computer-controlled pumps (Nemesys, Cetoni GmbH). 2.5 v/v% Pico-Surf surfactant (Sphere Fluidics, C024) was used in fluorinated HFE-7500 3M Novec.

### Bulk Activation Assay

Freshly isolated pDCs were incubated in 100 µL per 10^6^ cells PBA containing the IFNα Cytokine Catch Reagent (Miltenyi Biotec, 130-092-605) at 4 degrees Celsius for 20 minutes. Next, cells were washed and resuspended in X-Vivo 15 cell culture medium (Lonza), supplemented with 2% human serum (pooled; Sanquin), 1% antibiotics (penicillin-streptomycin), at 25.000 cells per 100 µL in U-bottom microwell plates. For intracellular IFNα stainings, cells were not pre-incubated with Cytokine Catch Reagent, but directly transferred to the microwells upon isolation.

### Single-Cell Activation Assay

Freshly isolated pDCs were incubated in 100 µL per 10^6^ cells PBA containing the IFNα Cytokine Catch Reagent (Miltenyi Biotec) at 4 degrees Celsius for 20 minutes. For the primed conditions, pDCs were primed with 500 U/mL IFNβ, at a concentration of 10^5^ cells per 100 µL in U-bottom microwell plates, prior to the incubation with IFNα Cytokine Catch Reagent. Next, cells were washed and resuspended in X-Vivo 15 cell culture medium (Lonza, BE02-060Q), supplemented with 2% human serum (pooled; Sanquin), 1% antibiotics (penicillin-streptomycin), at 2.6*10^6^ cells/mL for single-cell encapsulation in 92 pL droplets. Stimulus was dissolved in medium at twice the desired concentration to account for 2x dilution in the microfluidic device. For a list of al utilized stimuli, see **Supplementary Table 1**. For droplet production, flow rates of 900 µL/h for the oil phase and 300 µL/h for the aqueous phases were used. Droplet production and encapsulation rates were carefully monitored using a microscope (Nikon) at 10x magnification and a high-speed camera. The droplet emulsion was collected and covered with culture medium to protect droplets from evaporation. The encapsulated cells were incubated in Eppendorf tubes with a few punched holes to allow gas exchange, at 37 degrees Celsius and 5% CO2. After 18 hours of incubation, the droplets were de-emulsified by adding 100 µL 20 v/v% 1H,1H,2H,2H-Perfluoro-1-octanol (Sigma Aldrich, 370533) in HFE-7500.

### Antibody Staining

Cells were washed once with PBS and dead cells were stained with Zombie Green fixable viability dye (BioLegend, 423111), 1:10.000 in PBS, 100 µL) at 4 degrees Celsius for 20 minutes. Subsequently, cells were washed once with PBS and incubated with antibodies against surface proteins in 50 µL PBA at 4 degrees Celsius for 20 minutes. Next, cells were fixed and permeabilized with Cytofix/Cytoperm solution (BD Biosciences, 554714) at 4 degrees Celsius for 20 minutes. Cells were washed with Perm/Wash buffer (BD) and incubated with antibodies against intracellular proteins in 50 µL Perm/Wash buffer. For a full list of all utilized antibodies and reagents, see **Supplementary Table 2**.

### Flow Cytometry

Acquisition was performed in PBA on FACS Aria (BD Biosciences). Flow cytometry data were analyzed using FlowJo X (Tree Star). FMO stainings served as controls for gating strategy. For the gating strategy, the readers are referred to **Supplementary Figure 1**.

### Data Analysis and Statistics

Analysis and data visualization was performed using PRISM for windows version 9 (GraphPad). For statistical analysis, Mann-Whitney test, and Wilcoxon signed-rank test were performed. T-SNE multidimensional data analyses were performed using FlowJo X (Tree Star).

## Results

### Droplet-Based Microfluidics Allows for Single-Cell Activation of pDCs

*In vivo*, pDCs operate in complex microenvironments that influence their cellular behavior. One way to reveal this complexity is by studying pDCs in highly controlled microenvironments, enabled by utilizing innovative techniques and approaches such as droplet-based microfluidics. Cleverly designed microfluidic chips allow for the generation of thousands of droplets with high precision and control over the content of each individual droplet.

**Figure 1:**
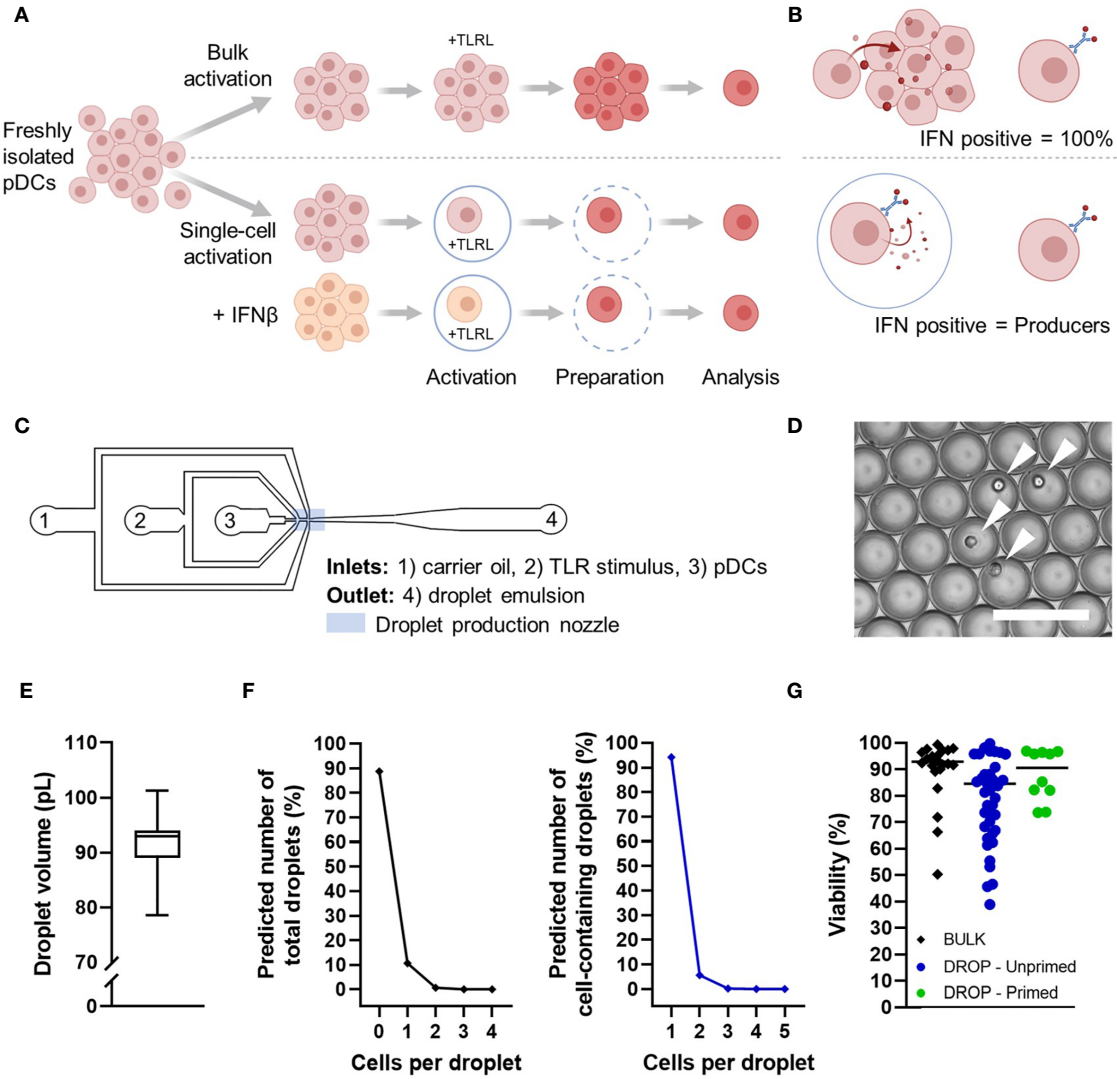
Schematic overview of experimental conditions and microfluidic device. **(A)** Human pDCs were isolated from buffy coats, primed with 500 U/mL IFNβ or left untreated. Next, all pDCs were coated with IFNα Catch Reagent and incubated with TLR ligands (TLRL) in bulk or droplets for 18 hours. Single cell encapsulation was achieved with concentrations of 1,300,000 cells/mL in 92 pL droplets on average. After incubation, encapsulated pDCs were retrieved from droplets by de-emulsification. Next, pDCs were washed thoroughly, fixed and permeabilized, stained for viability, extracellular IFNα, surface marker expression, intracellular granzyme B, and analyzed by flow cytometry. **(B)** Schematic representation of IFNα production in bulk vs in droplets. **(C)** Schematic overview of microfluidic chip with inlets (1–3), outlet (4), and droplet production nozzle in blue rectangle. **(D)** Microscopic image of the droplet emulsion with single-encapsulated pDCs indicated with white arrows. Scale bar equals 100 μm. **(E)** Box plot depicting droplet volumes in pL, n = 100 droplets. **(F)** Theoretical fractions of total droplets (left) and cell-containing droplets (right) with x number of cells per droplet, according to the Poisson distribution, average droplet size of 92 pL, and cell concentration of 1,300,000 million cells/mL. **(G)** Viability of analyzed cells per experimental condition.

In order to investigate the diversification of pDCs upon single-cell activation, freshly isolated human pDCs were either stimulated in bulk or individually in picolitre-sized droplets (**Figure 1A**). Briefly, pDCs were isolated from human buffy coats, primed with IFNβ or left untreated, and coated with cytokine Catch Reagent for the IFN-I readout. Therefore, during incubation, secreted IFNα was captured on the cells’ surface (**Figure 1B**). The concentrations used for stimuli and cytokines for IFN-I priming have been extensively tested, as described elsewhere (16). Additionally, the different concentrations used for the droplet conditions compared to the bulk conditions were considerably chosen to correct for the total amount of volume per cell, to ensure similar absolute quantities of stimuli across experimental conditions. Besides, throughout this study, IL-3 stimulation served as a control to assess the TLR-induced up/down-regulation of the markers of interest, while this cytokine can be considered as a growth factor which barely induces phenotypical or functional differentiation in pDCs (14).

A microfluidic device was used to encapsulate individual cells in water-in-oil droplets (**Figures 1C, D**), as previously optimized and described (16). The Tip-Loading method was applied for proper cell encapsulation (29). The produced droplet volumes ranged between 70 and 110 pL (**Figure 1E**). Since the encapsulation of cells in droplets is a random process following the Poisson distribution, theoretical fractions of total droplets and cell-containing droplets were calculated (**Figure 1F**). Single-cell encapsulation was ensured by using relatively low cell seeding densities that resulted in over 95% of cell-containing droplets with only one cell. After incubation, pDCs were retrieved from droplets by breaking the emulsion for additional down-stream analysis. Although the de-emulsification of water-in-oil droplets can be toxic to the cells, cell viability remained high throughout all procedures, allowing reliable analysis (**Figure 1G**).

Taken together, our droplet-based microfluidics platform allows for studying diversification of single TLR-activated pDCs in highly controllable microenvironments.

### Single-Cell Activation Limits the Production of IFN-Is and the Upregulation of Cytotoxicity Markers to Only Small Fractions of pDCs

Recent studies have demonstrated that bulk-activated pDCs analyzed at single-cell resolution show significant levels of cellular heterogeneity (10, 14). These results prompted us to explore the levels of diversification upon single-cell activation, focusing on IFNα production, the costimulatory molecule CD80, the cytotoxicity markers PD-L1 and TRAIL, the adhesion molecule CD2, and the secretion of granzyme B. Based on these markers, pDC subsets have been described in the past (1). In order to validate our single-cell findings, bulk-activated pDCs of matching donors served as an important control to literature and enabled us to assess the role of paracrine signaling in the upregulation of the markers of interest. In turn, IFN-I priming mimics the effects of paracrine signaling, but since the activation of the cells in these conditions occurs in droplets, the intrinsic behaviors are still elicited rather than the averaged outcomes that would occur in bulk-activated pDCs.

**Figure 2:**
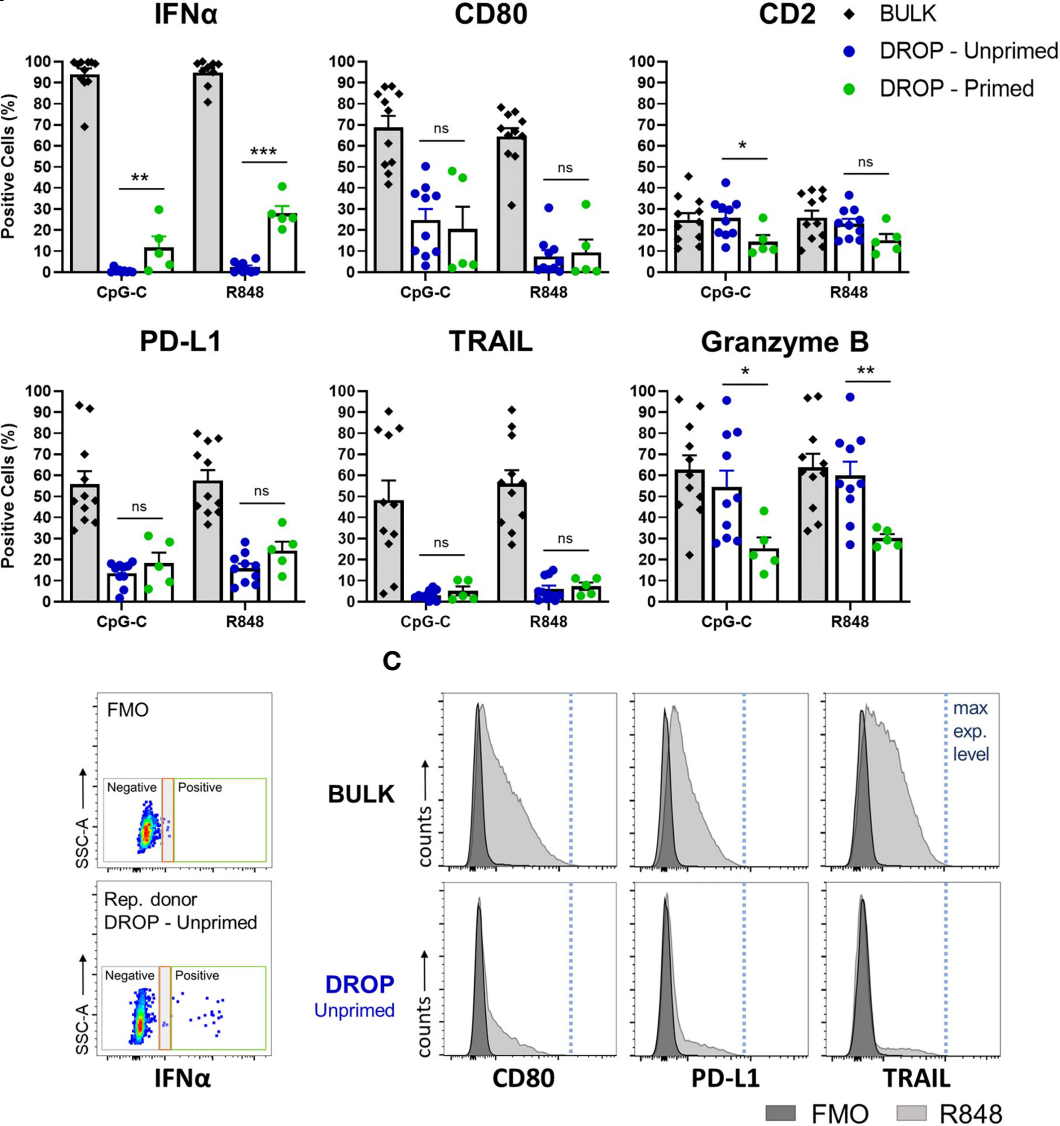
Single-cell analysis reveals functional and phenotypical heterogeneity within single-activated pDCs. Freshly isolated pDCs were either primed or left unprimed, coated with IFNα Catch Reagent, and activated in bulk or droplets as described before. The pDCs were activated with 5 or 50 μg/mL CpG-C or R848, for bulk and droplet conditions respectively, for 18h. Next, pDCs were stained for viability, IFNα secretion, marker expression of CD80, PD-L1, TRAIL, CD2, granzyme B, and analyzed by flow cytometry. **(A)** Data represent mean percentages of IFNα-secreting and marker-expressing pDCs plotted against treatment condition; error bars indicate SEM; BULK n = 11; DROP-Unprimed n = 10; DROP-Primed n = 5 for both CpG-C and R848. **(B)** Dot plots depicting IFNα expression levels of FMO control and representative donor, DROP-Unprimed. Depicted in the colored boxes are negative events (gray) technological noise (red), and positive events (green). **(C)** pDCs were treated as described above. The histograms are representatives of expression levels of viable pDCs stimulated with R848 in either bulk or droplets from one donor, compared to FMO controls. Indicated with the blue dotted line are the maximum expression levels. Mann–Whitney test *p < 0.05, **p < 0.01, ***p < 0.001. ns, non-significant.

In our experimental setup, bulk-activated pDCs reached up to 100% positivity for IFNα, as a result of the full saturation of IFNα binding to available Catch Reagents (**Figure 2A**). These IFNα molecules are produced by only a fraction of around 30% of IFNα-producing cells, according to numerous examples in literature (14). However, in droplets, the produced IFNα can only bind to the pDCs that have actually produced it. Therefore, the number of IFNα positive cells in the droplet conditions represent the actual IFNα producers. Notably, our results of unprimed single TLR-activated pDCs, representing the first responders, show that only a small fraction, of around 0.5-0.7%, of cells secrete IFNα (median for CpG-C; R848: 0.54; 0.76 respectively, *n* =10). Upon IFN-I priming, representing the second responders, percentages of IFN-producing pDCs increased up to 40%. This increase is the result of the IFN-I induced effects initiated during priming, which enhances the response rate upon activation compared to the numbers observed in naïve cells. This phenomenon is elicited upon both TLR7/8 (R848) and TLR9 (CpG-C) signaling, although the observed effect of IFN-I priming was higher for R848 activation (p = 0.0005) compared to CpG-C activation (p = 0.006).

In accordance with literature, bulk-activated pDCs show already levels of diversification upon TLR activation, with varying expression levels of CD80, PD-L1 and TRAIL (**Figure 2A**). Upon single-cell activation, the percentages of positive cells for these markers were significantly lower (p < 0.0001 for all markers, compared to bulk percentages). Interestingly, the effects of priming did not significantly affect the upregulation of these markers, indicating an underlying mechanism other than induced by IFN-Is. In contrast and in accordance with literature, the expression of CD2 remained relatively stable over time, independent of experimental conditions or stimulus (**Supplementary Figure 2**). Only priming seemed to have affected CD2 expression, with a significant reduction upon CpG-C activation. The differences were not significant when activated with R848. The secretion of granzyme B, assessed by intracellular staining, was similar for both the bulk and droplet-unprimed conditions, while priming significantly enhanced the secretion. The interpretation of these results was based on the finding that all pDCs that were not activated with TLR ligands expressed granzyme B, reaching up to 100% positivity (**Supplementary Figure 2**). This finding is in accordance with literature (30, 31).

The relatively low percentages of positive cells among the single TLR-activated pDCs, compared to the bulk-activated pDCs, raised the question whether these results were possibly due to technical limitations of the experimental setup. Therefore, we evaluated the expression levels of both IFNα and the individual markers from the single-activated pDCs, and compared these with the expression levels obtained from the FMO controls and bulk-activated pDCs. Despite the very low numbers of IFN-positive events in the unprimed droplet conditions, these events could be properly distinguished from technical noise, indicating that these events represent true IFN producers (**Figure 2B**). Besides, the expression levels of the markers of interest of single-activated pDCs reach up to those found for bulk-activated pDCs, indicating that the pDCs encapsulated in droplets can be properly activated and can induce their expression in a proper manner (**Figure 2C** and **Supplementary Figure 3**).

We conclude that single TLR-activated pDCs display a high degree of diversification, leading to heterogeneous outcomes, both functionally and phenotypically. Only small fractions secrete IFNα and induce the expression of surface markers CD80, PD-L1, and TRAIL. Moreover, IFN-I priming only increases the percentage of IFN producers and does not significantly affect the expression of cytotoxicity markers.

### PD-L1+/CD80- Subsets Are Specialized IFNα Producers and Express High Levels of TRAIL

While still most of the subset characterization is based on phenotypes, functional readouts are often correlated as a proof of concept and to reveal the importance, relevance and potential of the different subsets. Over the years, much progress has been made in identifying IFN-secreting subsets in the pDC compartment. One of the most recent studies identified and characterized 3 populations, with distinct phenotypes and functionalities, that arise upon activation in bulk. Based on the expression of PD-L1 and CD80, populations could be defined (14). PD-L1+/CD80- pDCs, referred to as P1, were found to be specialized in the production of IFN-Is and prevalent in patients with IFN-I-mediated autoimmune diseases. PD-L1-/CD80+ pDCs, referred to as P3, were found to promote T cell activation and T_h_2 differentiation. PD-L1+/CD80+ pDCs, referred to as P2, was not further addressed as being specialized in either IFN-I production or T cell activation.

**Figure 3:**
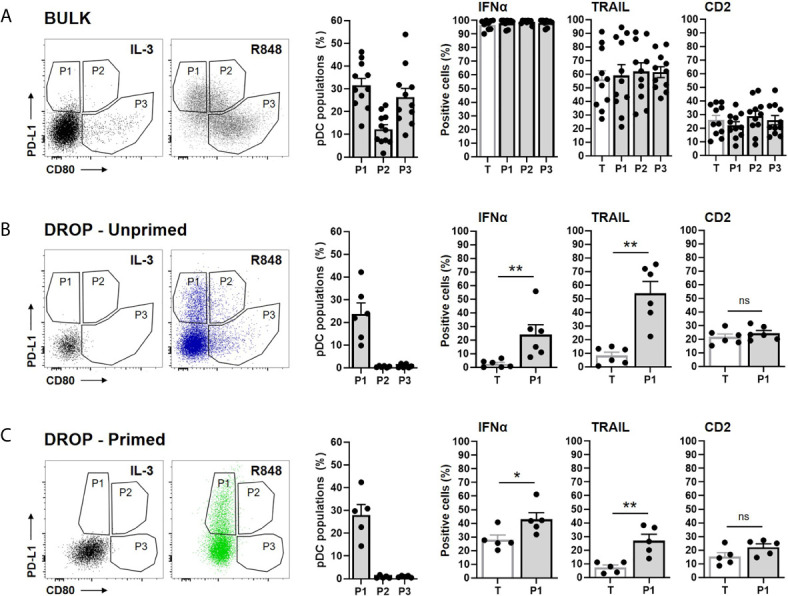
PD-L1+/CD80- subsets are specialized IFNα producers and express high levels of TRAIL. Freshly isolated pDCs were either primed or left unprimed, coated with IFNα Catch Reagent, and activated in bulk or droplets as described before. The pDCs were activated with 5 or 50 μg/mL R848, for bulk and droplet conditions respectively, for 18h. Next, pDCs were stained for viability, IFNα secretion, surface marker expression of CD80, PD-L1, TRAIL, CD2, granzyme B and analyzed *via* flow cytometry. **(A)** Based on the expression of PD-L1 and CD80, 3 populations were defined for the bulk activated pDCs. IL-3 stimulated pDCs served as a control for gating. The first histogram represents the percentages of the 3 populations. Other histograms represent the percentages of positive cells for the indicated markers, for the total viable pDCs (T) and the 3 populations (P1, P2, P3). **(B)** Data as in panel **(A)**, for unprimed single-activated pDCs. Expression of markers is only plotted for T and P1, because of the low percentages of P2 and P3 present in total. **(C)** Data as in **(B)**, for primed single-activated pDCs. Bars represent mean percentages; error bars indicate SEM; BULK n = 11; DROP-Unprimed n = 6; DROP-Primed n = 5. Mann–Whitney test *p < 0.05, **p < 0.01. ns, non-significant.

In this study, we set out to investigate whether we could find similar IFNα secretory profiles for P1, as described by Alculumbre et al. Therefore, bulk activated pDCs were plotted for PD-L1 and CD80 expression accordingly. Based on the expression levels obtained upon IL-3 stimulation, the 3 populations were defined (**Figure 3A**). Only the data for the R848-actived pDCs are shown, while for all CpG-C-activated pDCs similar results were found, across all experimental conditions (data not shown). Although our plots were slightly different from the data presented in Alculumbre et al., which could be explained by the different stimuli and incubation times used, we were able to define the 3 populations. Unfortunately, our experimental setup did not allow an interpretation regarding the IFNα readout, as described in prior results sections. The expression of TRAIL and CD2 did not significantly differ between the 3 different populations.

Next, the 3 populations were defined for the unprimed single-activated pDCs (**Figure 3B**). Interestingly, P2 and P3 were barely present, suggesting that the diversification into these 2 populations is dependent on paracrine and/or juxtacrine signaling. Because of the low abundance, the expression of IFNα, TRAIL and CD2 could not be properly examined in these two populations and therefore left out of the analysis. Hence, only P1, characterized by PD-L1+/CD80- was further characterized. Interestingly, in agreement with Alculumbre et al., we found that P1 expressed significantly more IFNα, compared to the total pDCs (T), implying that this population is specialized in IFNα production. Besides, the expression of TRAIL was also significantly higher in P1 compared to the total pDCs.

Finally, the 3 populations were defined for the primed single-activated pDCs (**Figure 3C**). Similar to the unprimed single-activated pDCs, P2 and P3 were barely present. A similar increase in IFNα-expressing and TRAIL expressing pDCs was observed for P1 as for the unprimed single-activated pDCs, although less potent.

A similar approach was undertaken for the CD2+ and CD2- pDCs. Although literature characterized the co-expression of IFN-Is, TRAIL and granzyme B for CD2+ cells, we couldn’t find any significant differences between the CD2+ and CD2- populations (data not shown) (10).

Together, in agreement with Alculumbre et al., our data demonstrate an increased IFNα signature for PD-L1+/CD80- pDCs, with high expression of TRAIL.

### Early IFN-Producing pDCs Share Phenotypical Characteristics Assigned to Previously Described Killer pDCs

The combination of IFN-I production and strong cytotoxic or antitumor activities has been described for (p)DCs in numerous studies (22–27). Therefore, we next zoomed in on the expression of cytotoxic markers on the fraction of early IFN-producing pDCs. For further analysis we used stringent gating conditions to correct for any possible technological noise that could arise from the binding of excessive IFNα to non-IFN-producing pDCs during the droplet de-emulsification phase, as described earlier.

**Figure 4:**
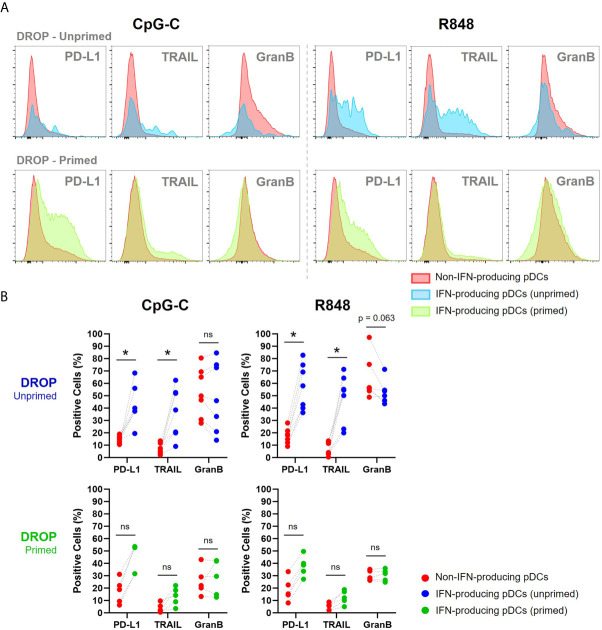
IFNα producing pDCs are phenotypically similar to previously described killer pDCs. **(A)** Single-encapsulated pDCs were either primed or left unprimed, incubated with IFNα Catch Reagent, activated with 50 μg/mL CpG-C or R848 for 18h and gated for IFN-producing (blue; unprimed, and green; primed) and non-IFN-producing pDCs (red). Histograms represent relative expression levels of PD-L1, TRAIL and granzyme B for merged data of 7 and 5 representative donors for unprimed and primed conditions, respectively. **(B)** Scatter plots show paired data of non-IFN-producing pDCs, IFN-producing pDCs and their corresponding percentage of positive cells for PD-L1, TRAIL and granzyme B per donor. Wilcoxon signed-rank test. *p < 0.05. ns, non-significant.

Our results show that early IFN-producing pDCs, those who produce IFN-Is in the unprimed conditions, express relatively high levels of PD-L1 and TRAIL compared to non-IFN-producing pDCs (**Figures 4A, B**). This finding is in line with the earlier described IFN-I-induced effect upon autocrine signaling that results in the upregulation of these markers, but it might as well be inherent to this specific fraction of pDCs (32, 33). Besides, considering the merged expression levels of all donors, the secretion of granzyme B seemed to be enhanced in IFN-producing pDCs compared to non-producing pDCs, though not significantly (**Figures 4A, B**).

For the populations of second responders, those who produce IFN-Is in the primed conditions, we found similar results, although less potent. This could be explained by the difference in secretion quantities between first and second responders, as literature suggests that first responders produce more cytokines than second responders due to multiple autocrine feedback loops (34, 35).

Altogether, first responders share phenotypical characteristics assigned to previously described IKpDCs, with relatively high expression of PD-L1 and TRAIL. The killer phenotype of the second responders, however, was not as evident as for first responders.

### Multi-Dimensional Data Analysis Shows Clustering of Early IFN-Producing pDCs With Co-Expression of PD-L1 and TRAIL

Next, we performed multidimensional data analysis to explore the possible existence of different pDC subsets. t-Distributed Stochastic Neighbor Embedding (t-SNE) is a data-visualization technique for dimensionality reduction that is particularly well suited for high-dimensional datasets. Since our panel consisted of only 6 dimensions (IFNα, CD80, PD-L1, TRAIL, CD2, and granzyme B), this technique is, in case of our study, more useful for visualizing co-expression of different markers and the indication of potential pDC subsets, rather than proving them.

**Figure 5:**
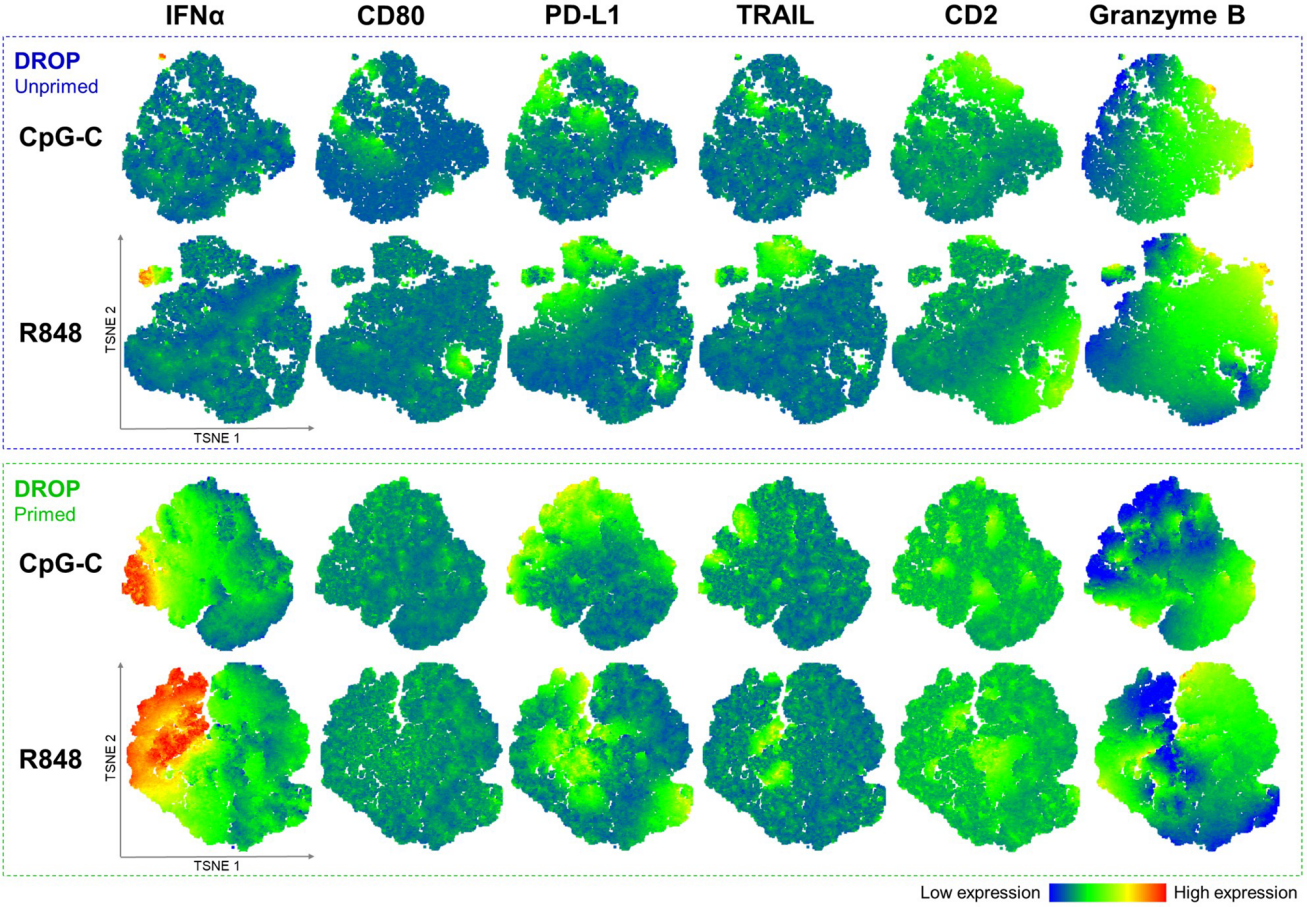
Multi-dimensional data analysis shows clustering of early IFNα-producing pDCs with co-expression of PD-L1 and TRAIL. Single pDCs were either primed or left unprimed, incubated with IFNα Catch Reagent, activated with 50 μg/mL CpG-C or R848 for 18 hours, retrieved, stained for viability and markers of interest, and analyzed with flow cytometry as described earlier. Depicted are t-SNE plots based on the reanalysis of earlier presented data, based expression of IFNα, CD80, PD-L1, TRAIL, CD2, and granzyme B; DROP-Unprimed n = 10; DROP-Primed n = 5. Individual events are colored according to the expression of each indicated marker. Corresponding heatmap indicates the level of expression from low (blue) to high (red). Iterations 1000, perplexity 30.

Data of all representative donors were combined for robustness of the analysis to generate the t-SNE plots. Notably, the unprimed single-TLR activated pDCs show that the first responders form a separate cluster, as visualized with the corresponding heatmaps, irrespective of the TLR ligand used for activation (**Figure 5**). These clusters of first responders showed relatively high levels of cytotoxic marker expression compared to the non-IFN producing cells. Interestingly, the t-SNE plots revealed high numbers of co-expression of the two cytotoxicity markers in this cluster (percentage PD-L1 positive events of TRAIL positive events: 88.9%). Overall, for both IFN producers and non-producers, the vast majority of TRAIL positive events co-expressed PD-L1 (co-expression numbers of total events; median ± median absolute deviation: 82.8% ± 6.5%, R848 activated, n = 7).

Similarly, t-SNE plots were generated for all primed single-TLR activated pDCs. Results show that primed IFN-producing pDCs, referred to as second responders, do no longer segregate in a separate population as we observed for the first responders (**Figure 5**). As previously shown, we confirmed that priming does not significantly affect the expression of PD-L1 and TRAIL, with t-SNE plots showing similar patterns of expression and co-expression numbers as compared to the unprimed data (co-expression numbers of total events; median ± median absolute deviation: 85.4 ± 2.6%, R848 activated, n = 5).

Overall, the multi-dimensional data analysis visualized by t-SNE plots showed that the unprimed IFN-producing pDCs form a separated cluster. These results indicate that the 1-3% of early IFN-producing pDCs are potentially a distinct subset with relatively high expression of PD-L1 and TRAIL.

### Early IFN-Producing pDCs Are Key Drivers for Orchestrating Population-Wide IFN-I Responses

Advancing single-cell technologies and refined experimental approaches have highlighted the multi-layered stochasticity present in the IFN-I system [reviewed in (36)]. This stochasticity turned out to be the underlying driver of the cellular heterogeneity that is observed today. Computational tools and mathematical models have helped interpret and understand the observed cellular heterogeneity and started to reveal the importance of first, second and non-responders involved in all kinds of biological processes. Therefore, we wanted to further characterize and investigate the role of first responders in eliciting population-wide effects.

**Figure 6:**
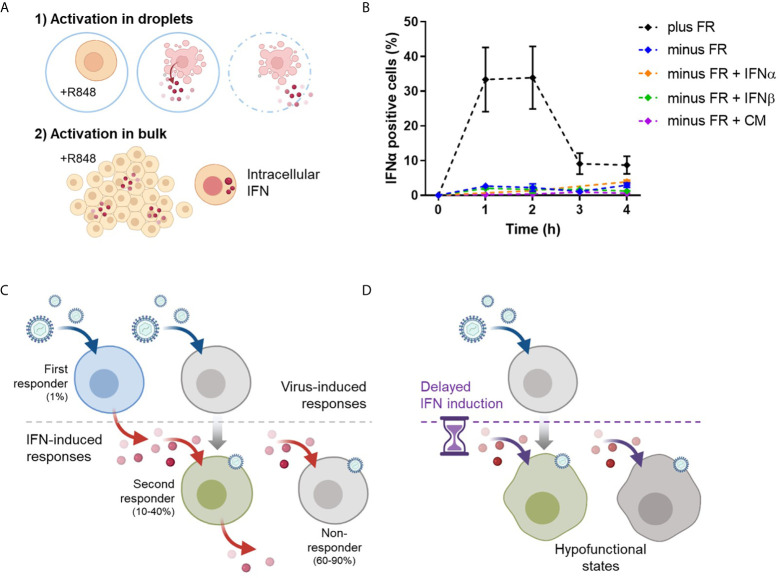
Timing of IFN-I dynamics is key for orchestrating population-wide IFN-I responses. **(A)** Freshly isolated pDCs were either only activated in bulk with 5 μg/mL R848 (2) or first activated in droplets with 50 μg/mL R848 (1), followed by the activation in bulk (2). In theory, activation 1) leads to the eradication of first responders (minus FR), whereas activation 2), without prior activation 1), will still include the first responders (plus FR). Next, cells were fixed, permeabilized and stained for intracellular IFNα over the course of the first 4 hours after bulk activation (2). **(B)** Depicted are the dynamics of IFNα positive cells per condition. The additives IFNα (100pg), IFNβ (250pg), and conditioned medium (CM, 100%) were added upon activation 2). Dots represent mean percentages; error bars represent SEM, n = 6. **(C)** Schematic overview of proper IFN-I induction by a small fraction of first responders, induced upon viral detection. Subsequently, the secreted IFN-Is induce the larger fraction of second responders, which arise from the population that did not became a first responder in the earlier phase. The majority is left unresponsive. **(D)** Without the proper timing of the IFN-induced responses by the first responders, pDCs will enter a hypofunctional state, as observed in panel **(B)**.

To assess population-wide IFN dynamics without the functional presence of the first responders, freshly isolated pDCs were first activated in droplets for 18h to eradicate the first responders (**Figure 6A**). During this incubation, the first responders will produce IFN-Is, after which they will enter an IFN-desensitized state (37–39). Next, all remaining pDCs, those that did not produce IFN-Is during the first activation, will receive a second activation in bulk. As a control, freshly isolated pDCs were directly activated in bulk to assess the IFN-I dynamics for the first crucial hours, measured by intracellular IFNα. These results show that within the first 4 hours the peak of IFN positive cells has passed, reaching up to 40% positivity (**Figure 6B**). This number corresponds with the second responders fraction described in prior result sections. In the absence of first responders, these levels of IFNα positive cells were not met. In fact, levels remained within the 1-3% range.

Following that, we assumed that the IFN-I response could be restored by supplying the approximate amount of IFN-Is that is usually produced by the first responders. Therefore, we added small amounts of IFNα or IFNβ to the conditions without the first responders present. Different amounts of IFN-Is were tested, ranging from 1 pg up to 1 μg (data not shown). However, none of it led to the increase in IFNα positive events. Finally, we added conditioned medium obtained from 1h bulk activation to the conditions without the first responders present, to ensure the right range of input for the second responders to get activated. Interestingly, also this approach did not led to the increase in IFNα positive events. This made us hypothesize that, under physiological conditions, population-wide IFN-I responses are driven by fractions of first responders, because of their fast and potent peak in IFNs that elicits the second responders (**Figure 6C**). When this trigger is delayed, the activated cells enter a hypofunctional state (**Figure 6D**).

To conclude, our results indicate that first responders can potentially serve as the key drivers of population wide IFN-I responses. In our experimental setup, IFN-I responses are impaired without their proper functioning.

## Discussion

The multifaceted biology of pDCs makes this versatile immune cell a promising therapeutic target for a wide range of diseases. The combination of their highly specialized function to produce massive amounts of IFNs, together with their ability to exert cytotoxic effector functions, makes pDCs the ideal treatment target to improve current immune therapies. Over the past decades, multiple different studies revealed heterogeneity present in the pDC compartment (10–14, 16). By changing the experimental approach from bulk activation to single-cell activation, we were able to further dive into the complex pDC biology by highlighting the important role for the first responders in eliciting population-wide IFN-I responses.

Similar to the phenomenon of a small fraction producing IFN-Is upon single TLR-activation, only small fractions of pDCs upregulate important cytotoxic markers, similar to the earlier described IKpDCs. It would be interesting to evaluate whether, besides their specialized function to secrete massive amounts of IFN-Is, IKpDCs share functional and phenotypical characteristics with NK cells, since several observations have suggested that these cells could represent a subset of NK cells (24). Additionally, our results are in agreement with previous studies that describe the effect of removing cell-to-cell communication resulting in a decreased expression of surface markers (16, 19). The phenomenon of a small fraction of cells amplifying population wide responses by activating surrounding cells *via* paracrine signaling has been extensively studied and proven for IFN-I signaling and multiple other immune reactions (17, 19, 40, 41). By making only a small fraction of cells respond to the initial stimulus, the response can be tightly regulated. For IFN signaling, this tight balance is crucial to avoid the harmful effects of excessive amounts of IFNs. For that matter, pDCs were classically described as being refractory to IFNα production upon repeated TLR activation, which could explain the non-responsiveness we observed in our study (42, 43). However, to the best of our knowledge, we are the first that have activated pDCs twice, of which the first activation happened in total isolation. Therefore, we can now conclude that this hypofunctional state is not initiated by soluble mediators released from the responding cells, but an intrinsic factor of pDC biology mediated by TLR-signaling.

Up to now, only HIV-stimulated pDCs allow for persistent IFNα production upon repeated stimulation (44). This persistent IFNα production was correlated with increased levels of IRF7 and was dependent upon the autocrine IFNα/β receptor feedback loop. Besides, multiple studies have proven the correlation between high basal expression levels of IRF7 with responsiveness, for both pDCs and other cell types (35, 45–48). IRF7 deficiency can even lead to recurrent influenza infection in humans, emphasizing its crucial role in eliciting proper IFN-I responses (49). This makes us hypothesize that the basal quantitative level of IRF7 present in single cells could potentially be one of the determining factors influencing cellular decision making, in particular whether to become a first or second responder, even though it has been stated that pDCs constitutively express background levels of IRF7 (50).

The multi-layered stochasticity underlying the IFN-I dynamics, thereby determining the first, second and non-responders, remains elusive. Though, the first responders seem to comprise a subset of high importance due to their dedication to the mass production of IFN-Is. Therefore, this subset is able to drive and orchestrate the population-wide IFN responses. In addition, possibly due to the autocrine signaling, the cytotoxic markers PD-L1 and TRAIL get upregulated, making them an ideal target for immune therapies. The question remains whether such hybrid cell will excel in performing both tasks *in vivo*. Although this subset might not excel in both, it could still be a crucial driver of all kinds of pDC functionalities. Therefore, a better understanding on the role of these highly specialized hybrid cells in orchestrating population-wide responses might push the field of pDC-based immune therapies to a new therapeutic level.

## Data Availability Statement

The raw data supporting the conclusions of this article will be made available by the authors, without undue reservation.

## Ethics Statement

Human primary pDCs were isolated from buffy coats of healthy donors (Sanquin), according to institutional guidelines and after informed consent per the Declaration of Helsinki. No additional ethics approval was required for their use, and written informed consent was provided by all donors.

## Author Contributions

LE, EC, NS, and JT designed the study. LE performed all experiments. LE analyzed the data. LE, EC and JT wrote the article. JT supervised the research. All authors contributed to the article and approved the submitted version.

## Conflict of Interest

The authors declare that the research was conducted in the absence of any commercial or financial relationships that could be construed as a potential conflict of interest.

## Abbreviations

IFN-I, type I interferon; IKpDC, interferon producing killer plasmacytoid dendritic cell; PBMC, peripheral blood mononuclear cell; pDC, plasmacytoid dendritic cell; PD-L1, programmed death-ligand 1; TLR, toll-like receptor; TLRL, toll-like receptor ligand; TRAIL, TNF-related apoptosis-inducing ligand.
