# Supplementary material for: Phenotypical Diversification of Early IFNα-Producing Human Plasmacytoid Dendritic Cells Using Droplet-Based Microfluidics
Source: Front Immunol. 2021 Apr 29;12:672729. doi: 10.3389/fimmu.2021.672729 (PMC8117785; doi:10.3389/fimmu.2021.672729)
Supplement: Supplementary file 1 [file DataSheet_1.docx]

Supplementary Material

# Supplementary Table 1:

| **Stimulus** | **Comment** | **Standard conc.** | **Standard conc.** | **Manufacturer** | **Catalog nr.** |
| --- | --- | --- | --- | --- | --- |
|  |  | **BULK** | **DROP** |  |  |
| R848 | Resiquimod | 5 μg/mL | 50 μg/mL | Enzo | ALX-420-038 |
| CpG-C | ODN M362 | 5 μg/mL | 50 μg/mL | Enzo | ALX-746-004-T100 |
| IL-3 | Recombinant human interleukin-3 | 1 ng/mL | 10 ng/mL | Peprotech | 200-03 |
| IFNβ | Recombinant human interferon beta | 500 U/mL |  | Peprotech | 300-02BC |
| IFNα2a | Recombinant human interferon alpha |  |  | Miltenyi Biotec | 130-093-874 |

**Supplementary Table 1.** Employed stimuli and cytokines.

# Supplementary Table 2:

| **Antigen** | **Clone** | **Label** | **Dilution** | **Manufacturer** | **Catalog nr.** |
| --- | --- | --- | --- | --- | --- |
|  |  |  |  |  |  |
| CD253 | RIK-2 | APC | 5 μL per 1M cells | Biolegend | 308209 |
| CD303 | 201A | APC | 5 μL per 1M cells | Biolegend | 354205 |
| CD80 | 2D10 | BV421 | 5 μL per 1M cells | Biolegend | 305221 |
| CD2 | RPA-2.10 | BV510 | 5 μL per 1M cells | Biolegend | 300217 |
| CD274 | 29E.2A3 | BV605 | 5 μL per 1M cells | Biolegend | 329723 |
| CD3 | HIT3a | FITC | 5 μL per 1M cells | Biolegend | 300305 |
| CD11c | 3.9 | FITC | 5 μL per 1M cells | Biolegend | 301603 |
| CD14 | 63D3 | FITC | 5 μL per 1M cells | Biolegend | 367115 |
| CD16 | 3G8 | FITC | 5 μL per 1M cells | Biolegend | 302005 |
| CD20 | 2H7 | FITC | 5 μL per 1M cells | Biolegend | 302303 |
| CD123 | 6H6 | FITC | 5 μL per 1M cells | Biolegend | 306013 |
| Granzyme B | QA16A02 | PE-Dazzle | 5 μL per 1M cells | Biolegend | 372215 |
| IFNα | LT27:295 | FITC | 1/30. | Miltenyi Biotec | 130-092-600 |
| Zombie Green |  | FITC | 1/1000 | Biolegend | 423111 |
| IFNα Secretion Assay |  | PE | 1/10. | Miltenyi Biotec | 130-092-605 |

**Supplementary Table 2: Employed antibodies and cytokine detection kit.**

# Supplementary Figure 1:


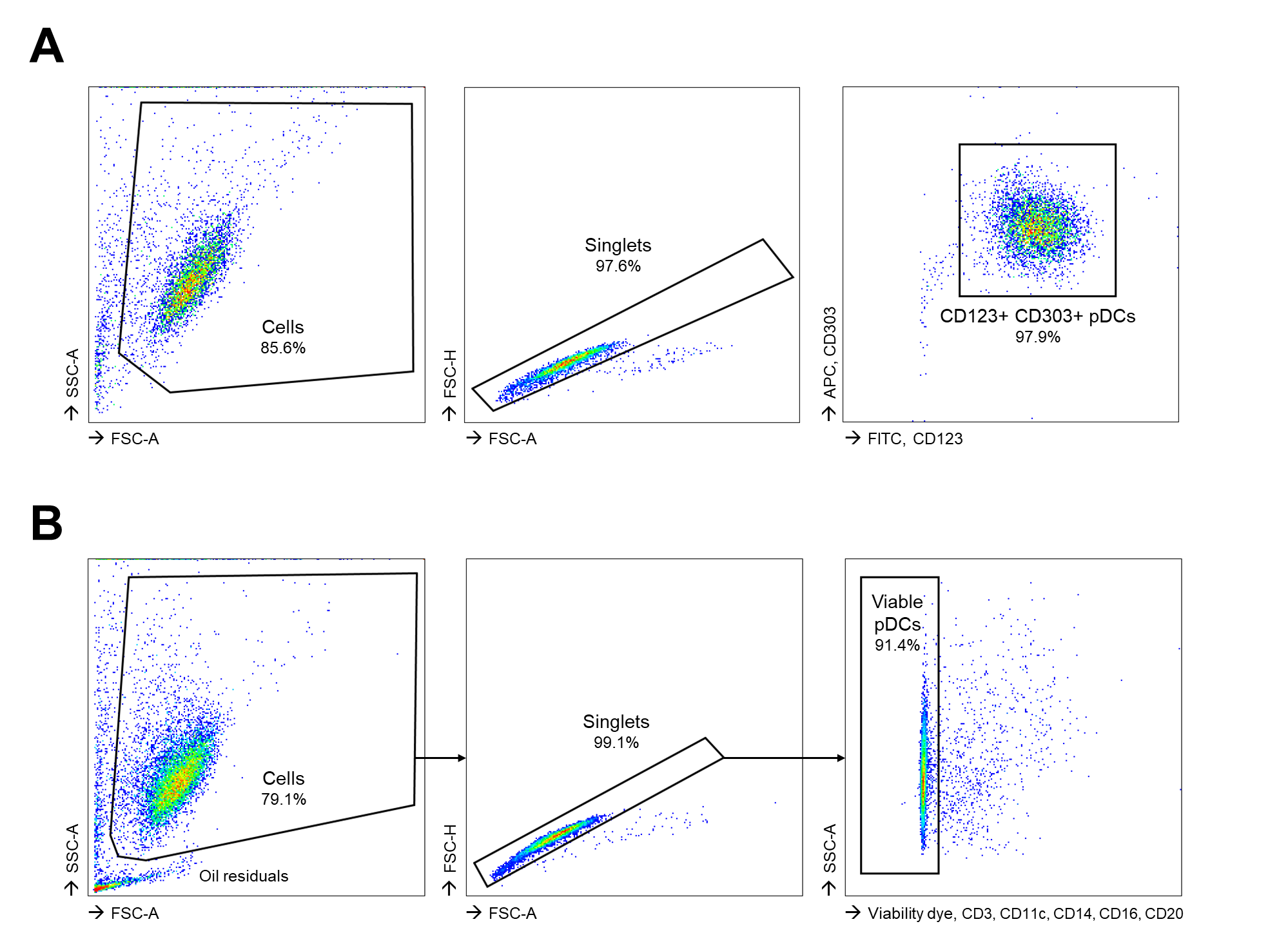


**Supplementary Figure 1: Gating Strategy. A** gating of pDC purity. **B** Gating of viable pDCs.

# Supplementary Figure 2:

**Supplementary Figure 2: Effects of IL-3 stimulation on marker expression.** Freshly isolated pDCs were coated with IFNα capture reagent, stimulated in bulk or encapsulated at a concentration of 1,300,000 cells/mL in 92 pL droplets. The pDCs were stimulated with 1 ng/mL or 10 ng/mL IL-3, for bulk and droplet conditions respectively, for 18h. After fixation and permeabilization, pDCs were stained for viability, IFNα secretion, surface marker expression of CD80, PD-L1, TRAIL, CD2, intracellular granzyme B (GranB), and analyzed via flow cytometry. Depicted are the mean values with SD.

# Supplementary Figure 3:


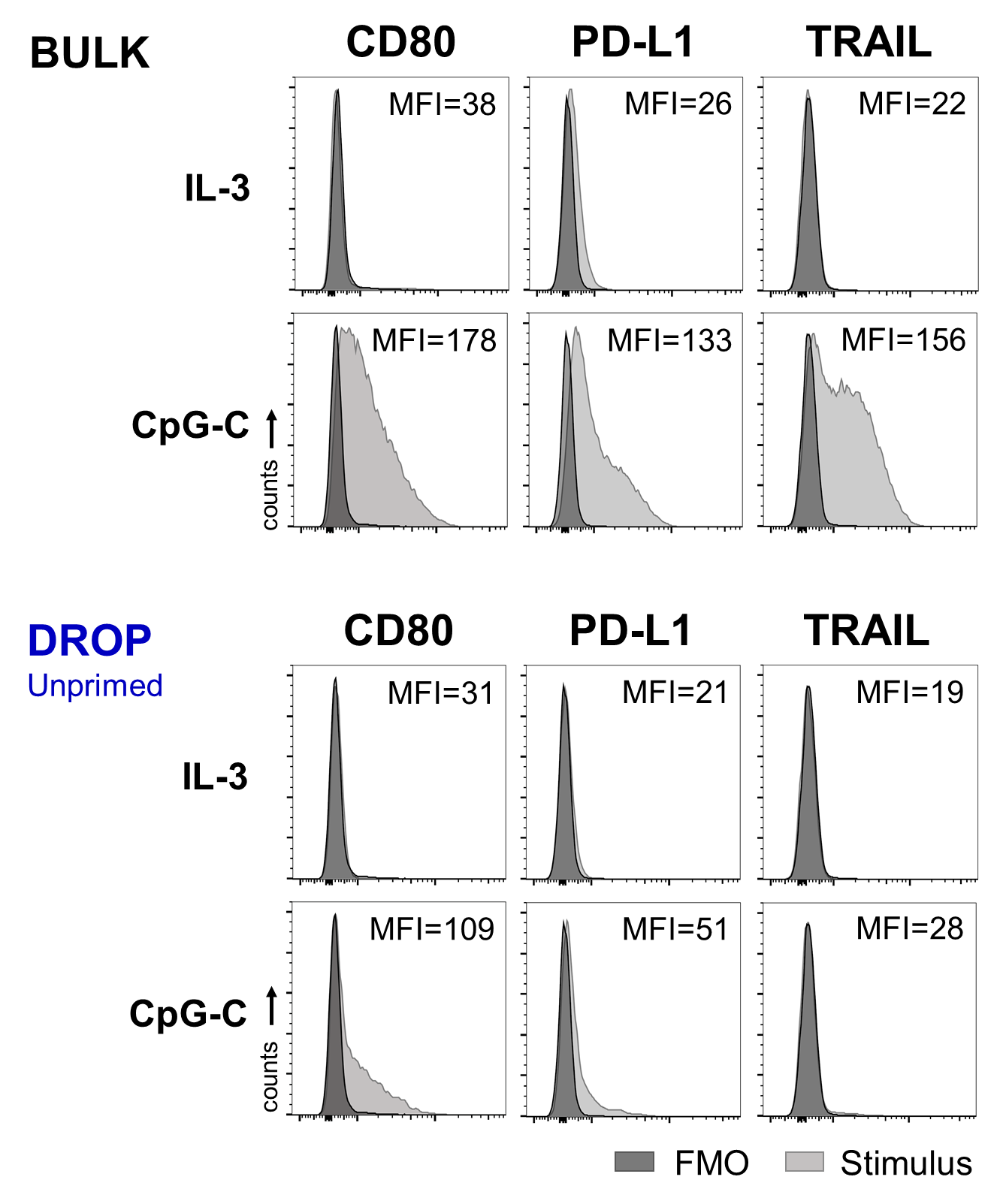


**Supplementary Figure 3: Expression levels for bulk conditions vs droplet conditions.** Freshly isolated pDCs were coated with IFNα capture reagent, stimulated in bulk or encapsulated at a concentration of 1,300,000 cells/mL in 92 pL droplets. The pDCs were stimulated with 1 ng/mL or 10 ng/mL IL-3, 5 μg/mL CpG-C 50 or μg/mL CpG-C, and 5 μg/mL R848 or 50 μg/mL R848, for bulk and droplet conditions respectively, for 18h. After fixation and permeabilization, pDCs were stained for viability, IFNα secretion, surface marker expression of CD80, PD-L1, TRAIL, intracellular granzyme b (GranB), and analyzed via flow cytometry. Shown are the expression levels of viable pDCs stimulated bulk (top) or droplets (bottom) from one representative donor, with corresponding MFI values, compared to unstained controls.
